# Diagnostic value of cystatin C in acute kidney injury among patients with sepsis: a systematic review and meta-analysis

**DOI:** 10.3389/fmed.2026.1769556

**Published:** 2026-06-10

**Authors:** Luping Cheng, Bo Zhang, Xia Hu, Wenxin Wang, Hong Wan, Jun Liao, Chuanliang Pan, Deqiong Xie

**Affiliations:** 1 Department of Intensive Care Unit, The Third People’s Hospital of Chengdu, Affiliated Hospital of Southwest Jiaotong University, Chengdu, China; 2 Department of Nephrology, Yibin Second People’s Hospital, Yibin, Sichuan, China

**Keywords:** acute kidney injury, biomarker, cystatin C, early diagnosis, sepsis

## Abstract

**Introduction:**

This study aimed to evaluate the diagnostic value of cystatin C (CysC) for the early detection of acute kidney injury (AKI) in patients with sepsis through a meta-analysis.

**Methods:**

Relevant studies were systematically retrieved from electronic databases using predefined search terms, including “sepsis,” “acute kidney injury,” and “diagnosis.” Study quality was assessed using the Quality Assessment of Diagnostic Accuracy Studies-2 (QUADAS-2) tool. A random-effects meta-analysis was conducted using Stata 12.0 software to calculate pooled effect estimates and corresponding 95% confidence intervals (CIs). Subsequently, a summary receiver operating characteristic (SROC) curve was constructed, and the area under the curve (AUC) was calculated to evaluate overall diagnostic performance.

**Results:**

A total of 17 studies on cystatin C for diagnostic testing were included, and a random-effects model was applied for meta-analysis. The results indicated that cystatin C exhibited good diagnostic performance for sepsis-associated acute kidney injury (SA-AKI), with an AUC of 0.88, sensitivity of 0.81, and specificity of 0.82. Subgroup analyses further demonstrated that studies based on the Sepsis 3.0 criteria showed higher specificity and diagnostic odds ratio (DOR), along with reduced heterogeneity. In addition, the combination of cystatin C with other biomarkers further improved diagnostic accuracy. Overall, these findings suggest that cystatin C is a promising biomarker for the early diagnosis of SA-AKI.

**Conclusion:**

This meta-analysis demonstrates that cystatin C has considerable value for the early diagnosis of SA-AKI. Although heterogeneity among included studies remains, the overall evidence supports its potential applicability in clinical practice.

**Systematic review registration:**

https://www.crd.york.ac.uk/PROSPERO/view/CRD420251128608, identifier PROSPERO (CRD420251128608).

## Introduction

Acute kidney injury (AKI) is a common and critical condition encountered across multiple clinical disciplines, characterized by a rapid onset that may lead to a marked deterioration in renal structure and function within hours to days. AKI not only results in a significant decline in glomerular filtration rate (GFR), leading to azotemia, but is also frequently accompanied by severe disturbances in fluid, electrolyte, and acid–base balance. In severe cases, multiple organ systems may be affected, resulting in serious complications such as acute pulmonary edema, hyperkalemia, and metabolic acidosis, thereby posing a substantial threat to patient survival and clinical outcomes.

According to previous epidemiological studies, the incidence of AKI among hospitalized patients ranges from 1 to 10% (1). In intensive care units (ICUs), this proportion increases markedly to 18–55%, with persistently high mortality rates; notably, the 90-day mortality rate may reach 41.9% (2). Furthermore, sepsis-associated acute kidney injury (SA-AKI) accounts for more than half of AKI cases in ICU patients. In addition, AKI not only prolongs hospital stay and increases healthcare costs but is also strongly associated with the subsequent development of chronic kidney disease (CKD) and end-stage renal disease (ESRD). It has been reported that approximately 30–70% of AKI survivors may progress to CKD.

Currently, the clinical diagnosis of SA-AKI primarily relies on two indicators: serum creatinine (Scr) and changes in urine output. From a physiological perspective, serum creatinine is a metabolic byproduct of muscle turnover, and its concentration is influenced by multiple non-renal factors, including muscle mass, age, sex, and dietary intake. However, following the onset of AKI, owing to the intrinsic renal functional reserve, serum creatinine and estimated glomerular filtration rate (eGFR) may not exhibit significant alterations even in the presence of substantial renal impairment. Consequently, this delayed response may hinder timely detection of AKI and result in missed opportunities for early therapeutic intervention (3). In addition, urine output is susceptible to various external influences, such as fluid administration and the use of diuretics, which may limit its ability to accurately reflect renal function and contribute to delays in early diagnosis. More importantly, in critically ill patients, sepsis and AKI may interact synergistically, leading to rapid clinical deterioration. Therefore, conventional diagnostic approaches demonstrate notable limitations in the early detection of AKI and in prognostic assessment, and may not adequately meet current clinical demands.

In recent years, with the continuous advancement of medical research, several novel biomarkers for AKI have been identified, including neutrophil gelatinase-associated lipocalin (NGAL), cystatin C (CysC), and kidney injury molecule-1 (KIM-1). These developments have provided new avenues for the early diagnosis and prognostic evaluation of AKI.

Among these biomarkers, CysC is a cysteine protease inhibitor with a molecular weight of approximately 13 kDa. It is consistently produced by all nucleated cells and is largely unaffected by factors such as muscle mass and age. Moreover, CysC is freely filtered by the glomerulus and subsequently almost completely reabsorbed and catabolized in the proximal tubules, thereby allowing for a more accurate reflection of changes in GFR (4–6). In addition, the measurement of CysC is relatively simple and cost-effective, making it suitable for rapid bedside assessment in intensive care settings and suggesting its potential for routine clinical use. Therefore, the present study conducted a meta-analysis to evaluate the diagnostic performance of CysC in the early detection of sepsis-associated acute kidney injury.

## Methods

### Search strategy and study selection

This systematic review was reported in accordance with the Preferred Reporting Items for Systematic Reviews and Meta-Analyses (PRISMA) guidelines (7). The PRISMA checklist is available in Supplementary material 1, and the protocol was prospectively registered with PROSPERO (registration number: [CRD420251128608]).

From database inception to October 1, 2025, a comprehensive literature search was conducted in PubMed, Embase, Scopus, Web of Science, and the Chinese Biomedical Literature Database (CBM). The detailed search strategy is presented in Supplementary material 2.

All retrieved records were imported into EndNote 21 (Clarivate Analytics) for initial deduplication using the built-in automated function. Subsequently, two independent reviewers (CLP and ZB) manually screened potential duplicates by comparing titles, authors, and publication years. Any discrepancies were resolved through discussion until consensus was reached.

Following deduplication, a dual-review process was implemented. Two reviewers (CLP and ZB) independently screened the titles and abstracts, and subsequently assessed full texts to identify studies meeting the inclusion criteria. In cases of disagreement, a third reviewer (HX) was consulted to achieve consensus. All data collection, extraction, and management were performed using Microsoft Excel (Microsoft Corporation).

### Inclusion and exclusion criteria

Inclusion criteria: (1) studies were limited to diagnostic accuracy studies, including prospective or retrospective cohort studies and case–control studies; (2) studies evaluating the association between SA-AKI and relevant biomarkers were included; (3) studies reporting at least one of the following diagnostic performance indicators were eligible: sensitivity, specificity, or area under the curve (AUC); and (4) participants were adults aged ≥18 years.

Exclusion criteria: (1) studies with incomplete data, data that could not be extracted or converted, or without accessible full texts; (2) studies involving pregnant populations; (3) case reports, animal studies, and review articles; and (4) non-original studies. In addition, if multiple studies conducted by the same authors involved overlapping populations, only the earliest published study or the study with the most comprehensive data was included.

### Data extraction and literature quality assessment

Data extraction was performed to collect the following information from each included study: basic study characteristics (first author, publication year, diagnostic criteria, sample source, and biomarker type), as well as the total number of participants. In addition, biomarker cutoff values, sensitivity, specificity, and AUC were extracted, and the Youden index was subsequently calculated. In cases of disagreement during data extraction, a third reviewer was consulted to resolve discrepancies through discussion. Furthermore, the methodological quality of the included studies was independently assessed by two reviewers (CLP and ZB) using the Quality Assessment of Diagnostic Accuracy Studies-2 (QUADAS-2) tool (8).

### Statistical analysis

Statistical analyses were performed using Stata version 12.0 (Stata Corp, College Station, TX, USA). Pooled effect estimates and their corresponding 95% confidence intervals (CIs) were calculated using a random-effects model (9), including sensitivity, specificity, and diagnostic odds ratio (DOR). The overall diagnostic performance was evaluated using the area under the summary receiver operating characteristic (SROC) curve. According to established criteria (10), the AUC values were interpreted as follows: 0.90–1.00, excellent diagnostic performance; 0.80–0.89, good performance; 0.70–0.79, moderate performance; 0.60–0.69, poor performance; and 0.50–0.59, no practical diagnostic value. Heterogeneity among studies was assessed using Cochran’s Q test and the *I*^2^ statistic. An *I*^2^ value of <30% was considered low heterogeneity, 30–50% moderate heterogeneity, and >50% substantial heterogeneity. Accordingly, a fixed-effects model was applied when heterogeneity was low (*I*^2^ ≤ 50%), whereas a random-effects model was used when heterogeneity was substantial (*I*^2^ > 50%). In this study, heterogeneity analyses for sensitivity and specificity revealed *I*^2^ values exceeding 50%. Moreover, diagnostic test accuracy studies often exhibit underlying clinical and methodological heterogeneity, including variations in study population characteristics, testing methodologies and platforms, cutoff values, and study designs. Accordingly, from a methodological standpoint, the random-effects model more appropriately accounts for inter-study variability and is generally considered the preferred approach for diagnostic meta-analyses. Publication bias was evaluated using Deeks’ funnel plots.

### Ethical considerations

This study did not involve the collection of primary data or the acquisition of identifiable personal information from participants. Therefore, in accordance with relevant guidelines for systematic reviews and meta-analyses, ethical approval was not required.

## Results

### Search results

**Figure 1:**
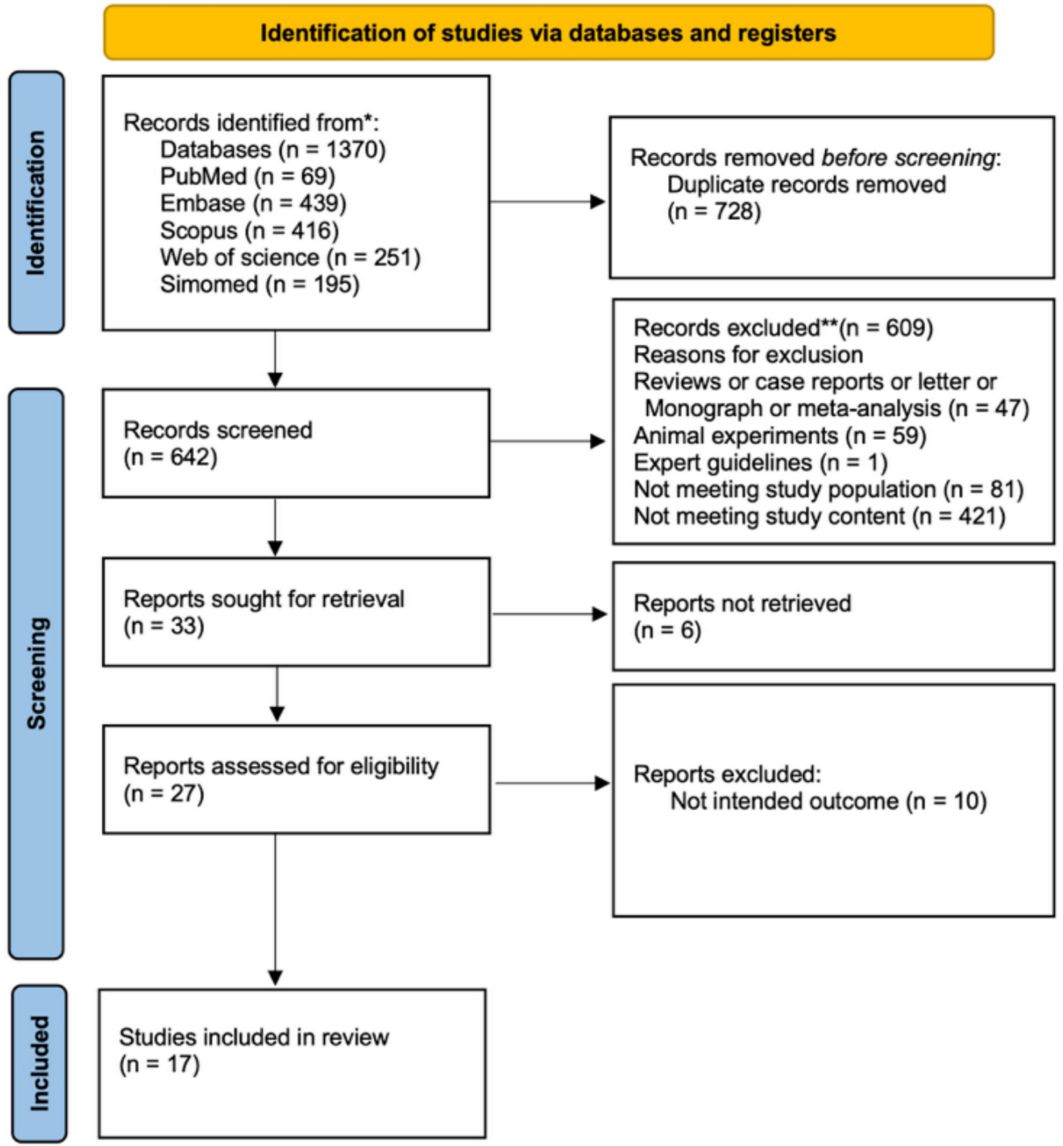
PRISMA flow diagram.

Based on the search results presented in Figure 1, a total of 1,370 records were initially identified. First, duplicate records were removed, resulting in the exclusion of 728 redundant publications. Subsequently, in accordance with the predefined inclusion and exclusion criteria, a preliminary screening of titles and abstracts was conducted, leading to the further exclusion of 606 records that did not meet the eligibility criteria. The remaining 27 studies were subjected to full-text assessment for eligibility. Following a comprehensive and systematic evaluation of the full texts, 17 studies were ultimately included in the final analysis, encompassing a total of 2,479 patients.

### Basic characteristics and quality evaluation of literature

**Figure 2:**
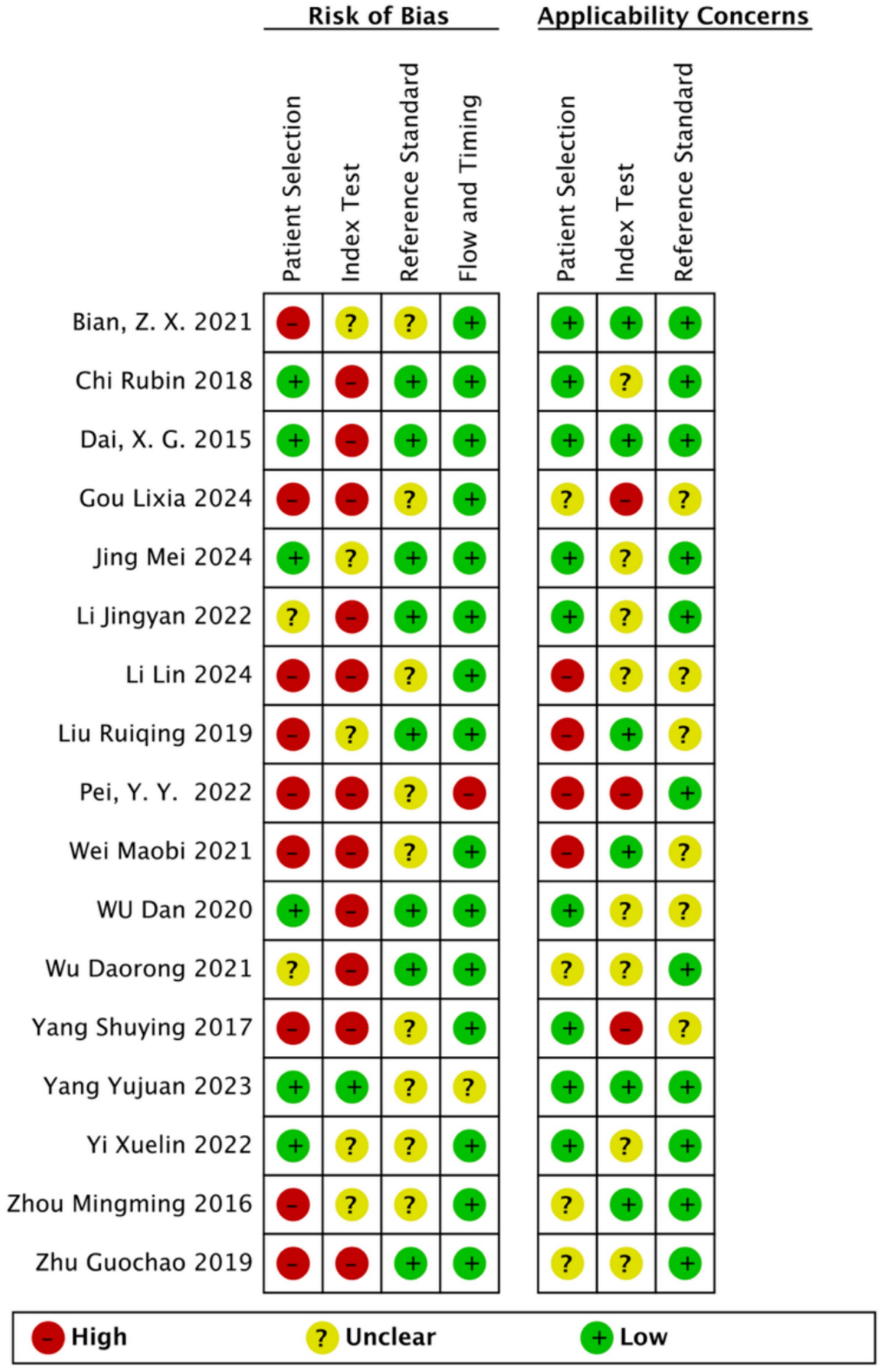
Risk of bias in included studies.

A total of 17 studies were included, among which 8 were prospective single-center investigations. The diagnostic performance of cystatin C, both as a standalone biomarker and in combination with other indicators, was systematically summarized, and relevant data were extracted accordingly. The corresponding results are presented in Tables 1, 2. Furthermore, study quality was assessed using the QUADAS-2 tool, as illustrated in Figure 2 and Supplementary Figure S1. According to the QUADAS-2 criteria, study quality was categorized as “low risk,” “high risk,” or “unclear risk of bias.” Based on the criteria for patient selection, 9 studies (11–19) were classified as “high risk,” whereas 6 studies (9, 20–24) were classified as “low risk.”

**Table 1:** Characteristics of studies about CysC included in the analysis.

Author	Year	Sepsis and AKI definition	Study design type	No. SA-AKI	No. non-AKI	Sample	Biomarker	AUC	Sen	Spe	Youden index	Cut-off value
Gou (18)	2024	Sepsis3.0, KDIGO	Retrospective cohort	114	48	Plasma	CysC	0.8700	0.8860	0.8120	0.6980	2. 228 ng/L
Li (17)	2024	Sepsis3.0, KDIGO	Retrospective cohort	82	216	Blood	CysC	0.7940	0.6250	0.8780	0.5030	1.220 mg/L
Jing (21)	2024	Sepsis3.0, KDIGO	Prospective cohort	71	122	Serum	CysC	0.8840	0.7995	0.7465	0.7180	1.720 mg/L
Yang (23)	2023	Emergency treatment guidelines for sepsis/septic shock in China (2018) (29), KDIGO	Retrospective cohort	67	53	Serum	CysC	0.8800	0.8509	0.8492	0.7001	1.640 mg/L
Pei (15)	2022	Sepsis3.0, KDIGO	Prospective cohort	60	102	Serum	CysC	0.8300	0.7670	0.8020	0.5690	10.400 ug/L
Li (30)	2022	Sepsis3.0, KDIGO	Retrospective cohort	74	158	Serum	CysC	0.7900	0.8140	0.8670	0.6810	1.560 mg/L
Yi (24)	2022	Surviving sepsis campaign: international guidelines for management of severe sepsis and septic shock: 2012, KDIGO	Retrospective cohort	51	66	Serum	CysC	0.8650	0.8430	0.8330	0.6760	1.140 mg/L
Wei (14)	2021	Sepsis3.0, KDIGO	Retrospective cohort	299	219	Serum	CysC	0.8830	0.7790	0.8810	0.6600	1.375 mg/L
Wu (31)	2021	Sepsis3.0, KDIGO	Retrospective cohort	40	62	Serum	CysC	0.8880	0.8750	0.8230	0.6980	1.950 mg/L
Bian (19)	2021	Sepsis3.0, KDIGO	Retrospective cohort	29	51	Serum	CysC	0.8930	NP	NP	-	NP
Wu (22)	2020	Sepsis3.0, KDIGO	Retrospective cohort	112	93	Serum	CysC	0.8870	0.7550	0.8890	0.6440	1.950 mg/L
Zhu (11)	2019	Sepsis3.0, KDIGO	Retrospective cohort	27	49	Blood	CysC	0.8770	NP	NP	-	NP
Liu (16)	2019	2012 SSC Guidelines for Sepsis, KDIGO	Retrospective cohort	42	47	Serum	CysC	0.7620	0.7700	0.7200	0.4900	2.560 mg/L
Chi (20)	2018	Surviving sepsis campaign: international guidelines for management of severe sepsis and septic shock: 2012; KDIGO	Prospective cohort	72	66	Serum	CysC	0.8280	0.7080	0.8030	0.5110	0.930 mg/L
Yang (13)	2017	NP; KDIGO	Prospective cohort	48	57	Serum	CysC	0.7820	0.9170	0.6140	0.5310	NP
Zhou (12)	2016	2012 SSCM Guidelines for Sepsis, KDIGO	Case–Control study	18	20	Serum	CysC	0. 7,650	0.7520	0.7790	0.5310	2.000 mg/L
Dai (9)	2015	The 2001 International Sepsis Definition Conference; KDIGO	Prospective cohort	55	57	Plasma	CysC	0.7370	NP	NP	-	NP

AUC, area under the curve of receiver operator characteristics. Sen, sensitivity. Spe, specificity, NP, not report. SSC, Surviving Sepsis Campaign. AKIN, acute kidney injury network. 1 μg/mL = 1 mg/L. 1 mg/L = 1,000,000 ng/L.

**Table 2:** Characteristics of studies about combined CysC and other biomarkers included in the analysis.

Author	Year	Sepsis and AKI definition	No. SA-AKI	No. Non-AKI	Sample	Biomarker	AUC	Sen	Spe	Youden index
Gou (18)	2024	Sepsis3.0, KDIGO	114	48	plasma	SOD + CysC + KIM-1	0.9480	0.8420	0.9370	0.7790
Li (17)	2024	Sepsis3.0, KDIGO	82	216	Venous blood	NGAL + CysC + BUN	0.9450	0.9024	0.8796	0.7820
Jing (21)	2024	Sepsis3.0, KDIGO	71	122	Serum	P16INK4a + IL-37 + CysC	0.9160	0.9396	0.8310	0.8820
Yang (23)	2023	Emergency treatment guidelines for sepsis/septic shock in China (2018) (29), KDIGO	67	53	Serum	HBP + CysC + PCT	0.9670	0.9102	0.9247	0.8349
Pei (15)	2022	Sepsis3.0, KDIGO	60	102	Serum	SCr + CysC	0.8470	0.9500	0.7000	0.6500
Li (30)	2022	Sepsis3.0, KDIGO	74	158	Serum	SCr + CysC + HDL-C	0.9300	0.9550	0.9660	0.9210
Yi (24)	2022	Surviving sepsis campaign: international guidelines for management of severe sepsis and septic shock: 2012, KDIGO	51	66	Serum	SCr + CysC + UmAlb	0.9130	0.8820	0.8330	0.7150
Wei (14)	2021	Sepsis3.0, KDIGO	299	219	Serum	APACHEII + CysC	0.9350	0.8860	0.8540	0.7400
Wu (31)	2021	Sepsis3.0, KDIGO	40	62	Serum	APACHEII + CysC + RBP	0.980	0.9750	0.8710	0.8460
Wu (22)	2020	Sepsis3.0, KDIGO	112	93	Serum	CysC + uNGAL	0.9840	0.9550	0.9630	0.9180
Liu (16)	2019	2012 SSC Guidelines for Sepsis, KDIGO	42	47	Serum	NGAL + KIM-1 + CysC	0.8910	0.9300	0.8700	0.8000
Chi (20)	2018	Surviving sepsis campaign: international guidelines for management of severe sepsis and septic shock: 2012; KDIGO	72	66	Serum	CysC + APACHEII	0.8800	0.7080	0.924	0.6320
Zhou (12)	2016	2012 SSCM Guidelines for Sepsis, KDIGO	18	20	Serum	Cys-C + uIL-18	0.9400	0.9030	0.8340	0.7370

AUC, area under the curve of receiver operator characteristics; Sen, sensitivity; Spe, specificity; RBP, retinol binding protein.

### Meta-analysis of CysC for the accuracy of SA-AKI diagnosis

**Figure 3:**
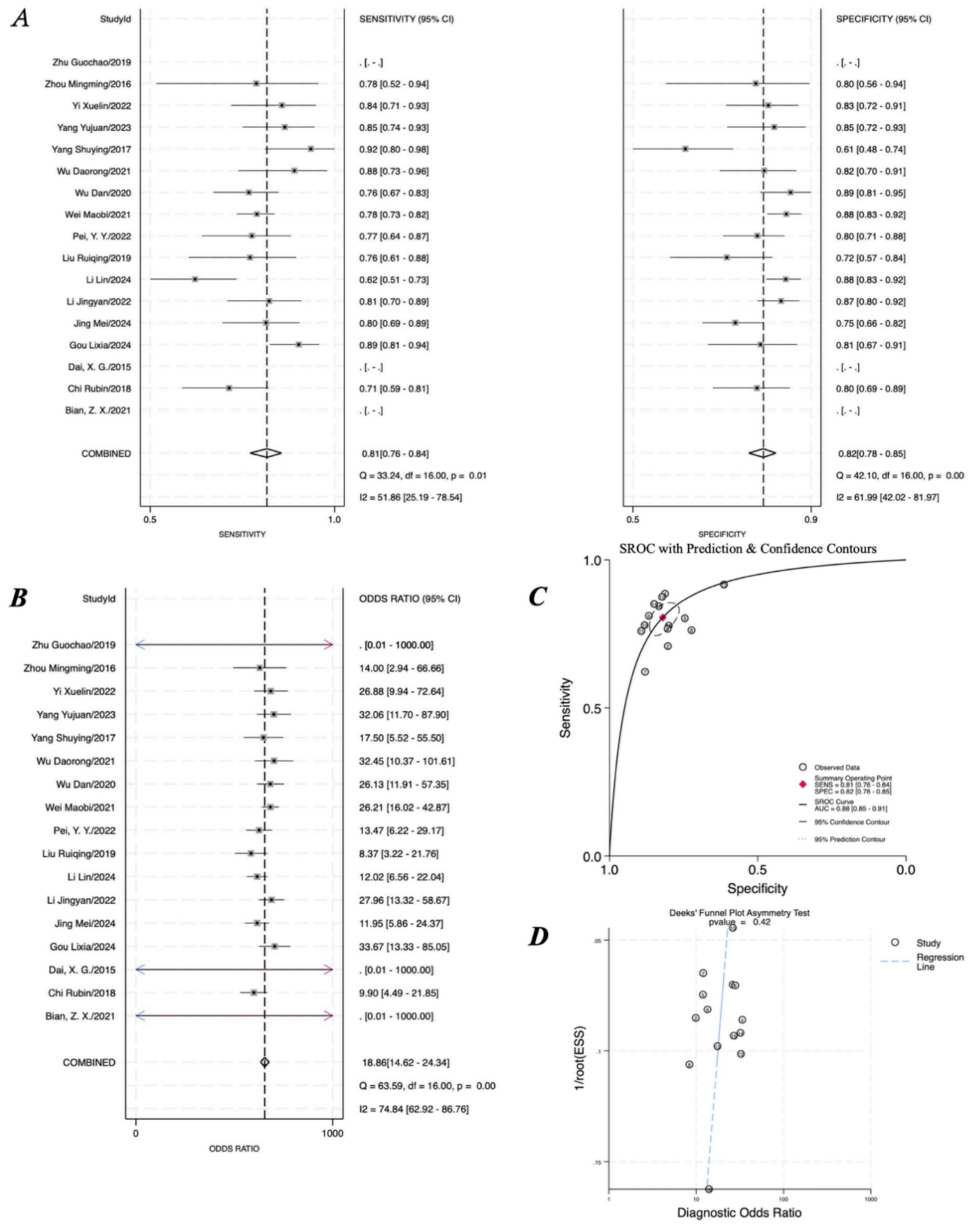
Diagnostic accuracy for Cys-C: **(A)** Diagnostic sensitivity and specificity; **(B)** diagnostic accuracy; **(C)** receiver operating characteristic curve (ROC); **(D)** publication bias.

**Figure 4:**
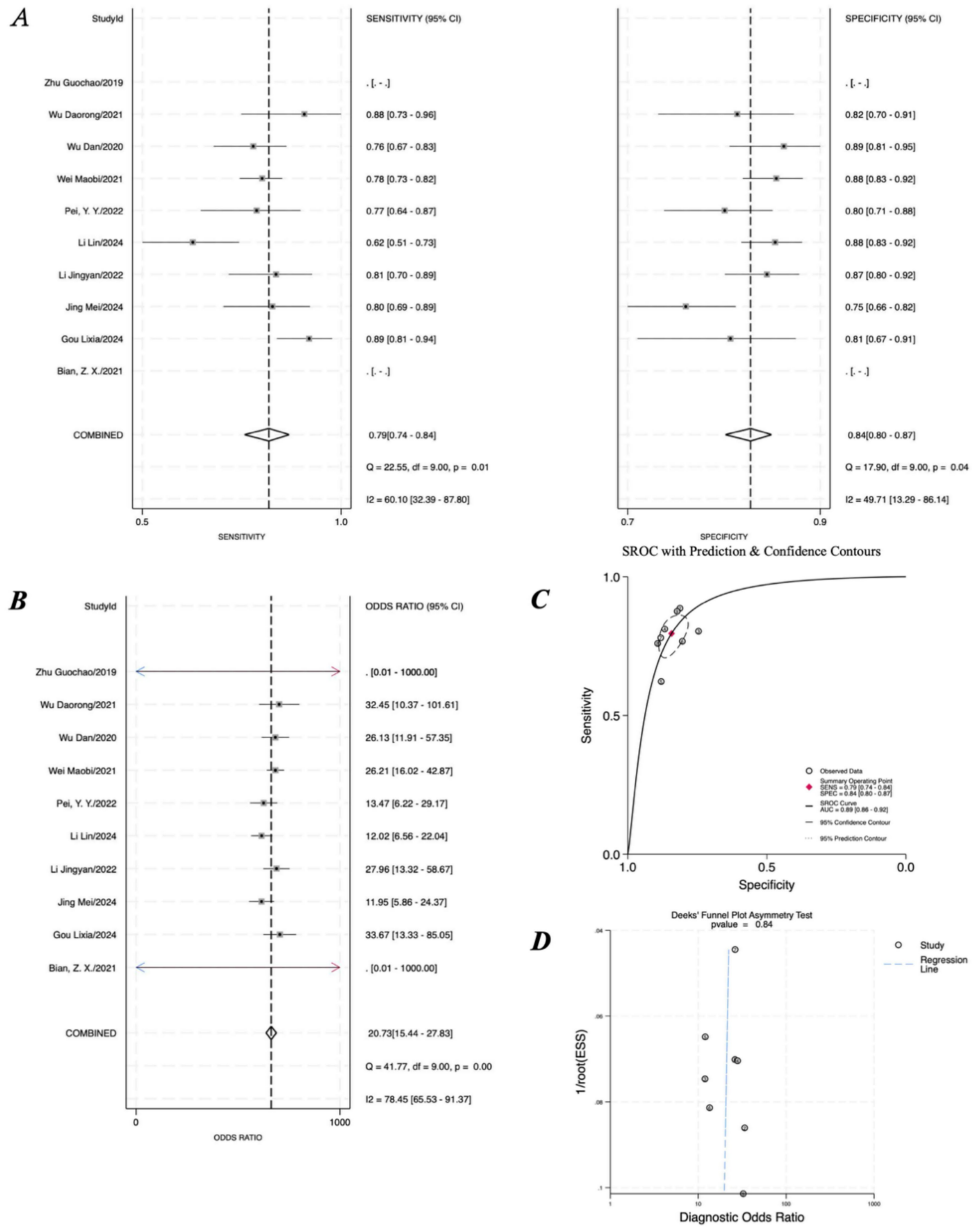
Diagnostic accuracy for Cys-C of Sepsis3.0: **(A)** Diagnostic sensitivity and specificity; **(B)** diagnostic accuracy; **(C)** receiver operating characteristic curve (ROC); **(D)** publication bias.

Based on the predefined inclusion and exclusion criteria, 17 studies evaluating the diagnostic accuracy of CysC for SA-AKI were included, of which 13 studies further assessed the performance of CysC in combination with other biomarkers. Subsequently, a meta-analysis was conducted using a random-effects model (25), with the corresponding results presented in Figures 3, 4. The pooled sensitivity, specificity, and DOR of CysC for diagnosing SA-AKI were 0.81 (95% CI: 0.76–0.84), 0.82 (95% CI: 0.78–0.85), and 18.86 (95% CI: 14.62–24.34), respectively (Figures 3A,B). In addition, the AUC was estimated to be 0.88 (95% CI: 0.85–0.91) (Figure 3C). According to the Deeks’ funnel plot asymmetry test, no significant publication bias was detected among the included studies (Figure 3D). However, heterogeneity analysis demonstrated moderate between-study variability. Specifically, the sensitivity forest plot yielded Q = 33.24, *p* = 0.01, and *I*^2^ = 51.86%, indicating moderate heterogeneity. Similarly, the specificity analysis showed a Q value of 42.10 (*p* < 0.001) and *I*^2^ = 61.99%, suggesting a moderately elevated level of heterogeneity.

To reduce heterogeneity arising from variations in sepsis definitions, a subgroup analysis was performed based on the Sepsis 3.0 criteria. The corresponding sensitivity, specificity, and DOR are presented in Figure 4. Following the subgroup analysis according to Sepsis 3.0, the pooled specificity increased to 0.84 compared with the overall estimate of 0.82, and the DOR increased to 20.73 relative to the overall value of 18.86, suggesting a more homogeneous and stable diagnostic performance within this subgroup. In addition, several studies have evaluated the diagnostic accuracy of Cys-C in combination with other biomarkers for SA-AKI (Table 2). The findings indicate that, compared with Cys-C alone, the combined application with additional biomarkers can significantly improve specificity. Meanwhile, according to the data presented in Table 1, the AUC for Cys-C in diagnosing SA-AKI ranges from 0.7 to 0.8, whereas the sensitivity of Cys-C combined with other biomarkers consistently exceeds 0.85.

Additionally, a Fagan nomogram analysis was performed (Supplementary Figure S2), demonstrating that at a pre-test probability of 30%, a positive test result increases the post-test probability to 66% (positive likelihood ratio [LR+] = 4), whereas a negative test result reduces the post-test probability to 9% (negative likelihood ratio [LR–] = 0.24).

### Heterogeneity analysis

Given the substantial heterogeneity observed in the meta-analysis, a meta-regression analysis was conducted to investigate potential sources of heterogeneity (Supplementary Table S1). Specifically, the following covariates were examined: publication year (before or after 2019), sample size (≥100 vs. <100), sepsis definition (Sepsis 3.0 vs. non-Sepsis 3.0), and the use of 1.5 mg/L as the diagnostic cutoff value (Supplementary Figure S2). Furthermore, subgroup meta-analyses were performed according to publication year, sample size, and cutoff value (Supplementary Figures S3–S8). Although heterogeneity appeared to be reduced in studies published before 2019, the number of included studies in this subgroup was limited. Therefore, these analyses did not demonstrate a statistically significant reduction in overall heterogeneity.

### Analysis of other indicators on the accuracy of SA-AKI

This study included 17 articles, all of which evaluated the diagnostic performance of biomarkers other than cystatin C (or cystatin C in combination with other indicators) for SA-AKI (Supplementary Table S2). The studies were screened according to predefined criteria, namely an area under the receiver operating characteristic curve (AUC) > 0.9 and a Youden index >0.8. Based on these criteria, four studies were ultimately selected to further elucidate the potential role of these biomarkers in the diagnosis of SA-AKI. The biomarkers identified included P16INK4a, interleukin-37 (IL-37), heparin-binding protein (HBP), procalcitonin (PCT), SCr, high-density lipoprotein cholesterol (HDL-C), and urinary neutrophil gelatinase-associated lipocalin (uNGAL).

## Discussion

The clinical manifestations of sepsis range from localized infection to severe systemic inflammatory response syndrome, often involving multiple organ systems and representing a substantial global health burden. AKI is a common and severe complication of sepsis, and the two conditions interact bidirectionally, exacerbating disease progression. This interaction is associated with an increased risk of complications, poorer clinical outcomes, and elevated mortality in patients with sepsis. Accordingly, in the present meta-analysis, 17 studies were included to systematically evaluate the diagnostic performance of CysC, both alone and in combination with other biomarkers. Overall, the findings indicate that CysC exhibits a pooled sensitivity of 0.81 and specificity of 0.82 for the diagnosis of SA-AKI, with a DOR of 18.86 and an AUC of 0.88, suggesting a high level of overall diagnostic accuracy. However, moderate to high heterogeneity was observed across the included studies, indicating that these findings should be interpreted with caution and warrant further investigation.

First, compared with Scr, CysC is relatively stable and less influenced by inflammatory states, infection severity, or fluctuations in muscle mass. Its levels increase during the early stages of renal dysfunction, enabling more rapid and accurate detection of subtle changes in GFR (26). In the context of sepsis, when renal hypoperfusion, microcirculatory dysfunction, or early alterations in GFR occur, elevations in CysC frequently precede increases in Scr, thereby conferring a distinct advantage for the early detection of SA-AKI. This characteristic renders CysC particularly suitable for critically ill patients, especially those with muscle wasting or hemodynamic instability. However, it should be noted that CysC levels may also be influenced by non-renal factors, including thyroid dysfunction, glucocorticoid therapy, smoking, and systemic inflammatory conditions (27, 28). Such factors may contribute to inter-study variability in baseline CysC levels, thereby partially accounting for the heterogeneity observed across studies.

To further investigate the moderate-to-high heterogeneity observed in this study, subgroup analyses were conducted based on several variables, including sample size, cutoff values, and year of publication. The results suggest that these factors may partially influence diagnostic performance. However, due to the limited number of studies within certain subgroups, the corresponding estimates may be unstable. In the subgroup analysis stratified by sepsis definitions, studies employing the Sepsis 3.0 criteria demonstrated relatively higher and more consistent diagnostic performance metrics. This observation may be attributable to the fact that Sepsis 3.0 places greater emphasis on organ dysfunction and applies more stringent assessment criteria, thereby partially reducing heterogeneity in patient disease course and severity. Nevertheless, given the still limited number of studies in the relevant subgroups, these results should be interpreted with caution. Additionally, this study explored potential sources of heterogeneity through meta-regression analysis. Considering the relatively small number of included studies in conjunction with a large number of covariates, there exists a risk of model overfitting. Therefore, the meta-regression findings should be regarded as exploratory, primarily intended to identify potential influencing factors rather than as definitive conclusions.

Notably, both sensitivity and specificity forest plots demonstrated moderate heterogeneity (*I*^2^ > 50%). The potential sources of this heterogeneity are analyzed as follows: (1) differences in detection methodologies across studies; (2) inconsistent timing of measurements—given the rapid progression of SA-AKI, variation in sampling windows may result in divergent directions and magnitudes of biomarker changes; (3) variability in patient characteristics, including the use of mechanical ventilation or differing levels of hemodynamic support; and (4) the absence of standardized cutoff values. Future investigations should aim to establish clinically actionable, platform-specific recommended thresholds.

In this study, we evaluated not only the diagnostic performance of CysC as a single biomarker for SA-AKI but also its potential utility in combination with other biomarkers. It is important to note that the analysis of CysC combined with other markers was limited to qualitative synthesis rather than quantitative meta-analysis. Quantitative pooling of combined indicators was not feasible for several reasons. First, the combinations of biomarkers employed across studies varied substantially (e.g., NGAL, KIM-1, PCT, HDL-C, APACHE II), with no standardized combination protocol. Second, significant heterogeneity existed in detection methods, cutoff values, and timing of measurements. Furthermore, some studies did not fully report key diagnostic parameters, such as sensitivity and specificity, resulting in inconsistent data structures. Collectively, these factors generated substantial clinical and methodological heterogeneity, rendering quantitative meta-analysis both impractical and statistically unsound. Nevertheless, the included studies generally suggest that combining CysC with biomarkers reflecting distinct pathophysiological mechanisms may enhance diagnostic performance for SA-AKI. For example, combined testing with NGAL and KIM-1, which reflect tubular injury, or with inflammation-related markers such as IL-37 and PCT, demonstrated high AUC values (>0.90) and favorable sensitivity and specificity in some studies. In principle, such multi-marker strategies can mitigate the limitations inherent to individual markers, providing more comprehensive pathophysiological insight. However, it should be emphasized that these conclusions are primarily based on descriptive summaries of individual study findings and do not constitute quantitative evidence validated by meta-analysis. Accordingly, these results should be interpreted cautiously and regarded as exploratory, indicating potential directions for future research rather than definitive conclusions. Future investigations should focus on high-quality, multicenter studies utilizing standardized biomarker panels, harmonized testing methodologies, and consistent reporting standards. Such efforts would facilitate future quantitative meta-analyses of combined biomarkers and further elucidate their clinical utility in the early diagnosis of SA-AKI.

Further analysis using Fagan’s nomogram revealed that, at a pre-test probability of 30%, a positive CysC result increases the post-test probability to 66% (LR^+^ = 4), whereas a negative result decreases it to 9% (LR^−^ = 0.24), demonstrating CysC’s robust risk reclassification performance. These findings provide critical guidance for clinical assessment of disease progression, early initiation of fluid management, and implementation of individualized renal protection strategies.

In addition, we reviewed diagnostic studies of other non-CysC biomarkers reported in the literature. Several high-performance biomarkers meeting the criteria of AUC > 0.9 and Youden index > 0.8 were identified, including P16INK4a, IL-37, HBP, PCT, SCr, HDL-C, uNGAL, among others, highlighting their potential utility in early detection and risk stratification.

This study presents several limitations that should be acknowledged when interpreting the findings. First, the meta-analysis included several retrospective studies. Although all included studies adopted the internationally standardized KDIGO criteria for AKI diagnosis, thereby ensuring endpoint consistency, retrospective study designs are inherently susceptible to selection and information biases, which may influence the heterogeneity of the pooled results. Owing to the limited number of retrospective studies and incomplete data in some cases, subgroup analyses based on study design, as well as sensitivity analyses by sequentially excluding retrospective studies, could not be performed. Consequently, readers are advised to interpret the pooled results with caution. Second, certain subgroups included a limited number of studies—often only three to four articles—which resulted in unstable effect estimates and may have led to overestimation or bias in intergroup comparisons. Some included studies were rated as having a high risk of bias in the “patient selection” domain of the QUADAS-2 tool, primarily due to case–control designs or non-consecutive enrollment. Such selection strategies may introduce selection bias, potentially inflating sensitivity and overall diagnostic performance. Furthermore, in studies utilizing healthy populations as controls, specificity may also be artificially elevated. Therefore, the pooled diagnostic performance results should be interpreted with caution. Third, this study could not evaluate the diagnostic and predictive performance of cystatin C in subgroups stratified by shock severity or by the presence of persistent acute kidney injury. This limitation arose primarily from the general lack of stratified data for these clinical subgroups in the original studies; most studies reported only overall diagnostic accuracy. Moreover, the ability of baseline cystatin C to predict persistent or worsening AKI remained unassessed, as the included studies were predominantly diagnostic in nature, focusing on AKI occurrence rather than its subsequent progression, and lacked longitudinal follow-up data as well as standardized definitions of prognostic outcomes. Finally, most studies were single-center, small-sample prospective cohort investigations, with substantial variability in study design, patient selection criteria, sampling time points, and testing platforms, further exacerbating heterogeneity in cross-study comparisons. Additionally, inconsistent definitions of sepsis were employed across studies. Although studies utilizing Sepsis 3.0 demonstrated more stable diagnostic performance, some investigations continued to apply earlier definitions, leading to discrepancies in disease severity and organ injury profiles among patient cohorts. Furthermore, several studies did not adequately control for confounding factors influencing CysC levels, potentially compromising the precision of diagnostic performance evaluations.

Despite these limitations, the present study underscores the considerable potential of CysC in early screening and risk stratification of SA-AKI. First, large-scale, multicenter prospective investigations strictly adhering to the Sepsis 3.0 definition are warranted to minimize structural heterogeneity arising from variable disease definitions and to enhance the generalizability and reproducibility of research findings. Second, standardization of testing procedures and methodologies is essential. This includes harmonizing CysC detection platforms, calibration systems, and recommended diagnostic thresholds, which will facilitate the determination of optimal cutoff values across diverse clinical settings and thereby improve diagnostic accuracy. Concurrently, multi-biomarker combination strategies—such as co-assessing CysC with NGAL, TIMP-2·IGFBP7, KIM-1, and other markers indicative of tubular injury, inflammation, or cell cycle arrest—have the potential to substantially enhance diagnostic performance. Therefore, future efforts may focus on developing comprehensive predictive models or scoring systems based on multiple biomarkers, integrated with machine learning or artificial intelligence algorithms, to achieve more precise SA-AKI risk stratification and personalized early warning. Notably, subsequent research could further investigate the dynamic trajectories of CysC across SA-AKI stages—including early trend shifts, doubling time, and correlations with fluid responsiveness and renal microcirculatory injury—potentially informing more targeted and timely therapeutic interventions.

## Conclusion

This study demonstrates that cystatin C possesses considerable early diagnostic utility for SA-AKI. Although heterogeneity remains evident across individual studies, the cumulative evidence supports its potential applicability in clinical practice.

## Data Availability

The original contributions presented in the study are included in the article/Supplementary material, further inquiries can be directed to the corresponding authors.
