# Supplementary material for: Diagnostic value of cystatin C in acute kidney injury among patients with sepsis: a systematic review and meta-analysis
Source: Front Med (Lausanne). 2026 Jun 10;13:1769556. doi: 10.3389/fmed.2026.1769556 (PMC13290599; doi:10.3389/fmed.2026.1769556)
Supplement: Supplementary file 2 [file Supplementary_File_2.DOCX]

Pubmed

#1 "Sepsis"[Mesh]

#2 (((((((((((((((Bloodstream Infection[Title/Abstract]) OR (Bloodstream Infections[Title/Abstract])) OR (Infection, Bloodstream[Title/Abstract])) OR (Septicemia[Title/Abstract])) OR (Septicemias[Title/Abstract])) OR (Blood Poisoning[Title/Abstract])) OR (Blood Poisonings[Title/Abstract])) OR (Poisonings, Blood[Title/Abstract])) OR (Poisoning, Blood[Title/Abstract])) OR (Severe Sepsis[Title/Abstract])) OR (Sepsis, Severe[Title/Abstract])) OR (Pyemia[Title/Abstract])) OR (Pyemias[Title/Abstract])) OR (Pyaemia[Title/Abstract])) OR (Pyohemia[Title/Abstract])) OR (Pyohemias[Title/Abstract])

#3 #1 OR #2

#4 "Acute Kidney Injury"[Mesh]

#5 ((((((((((((((((((((((Acute Kidney Injuries[Title/Abstract]) OR (Kidney Injuries, Acute[Title/Abstract])) OR (Kidney Injury, Acute[Title/Abstract])) OR (Acute Renal Injury[Title/Abstract])) OR (Acute Renal Injuries[Title/Abstract])) OR (Renal Injuries, Acute[Title/Abstract])) OR (Renal Injury, Acute[Title/Abstract])) OR (Kidney Failure, Acute[Title/Abstract])) OR (Acute Kidney Failures[Title/Abstract])) OR (Kidney Failures, Acute[Title/Abstract])) OR (Acute Kidney Failure[Title/Abstract])) OR (Acute Renal Failure[Title/Abstract])) OR (Acute Renal Failures[Title/Abstract])) OR (Renal Failures, Acute[Title/Abstract])) OR (Renal Failure, Acute[Title/Abstract])) OR (Renal Insufficiency, Acute[Title/Abstract])) OR (Acute Renal Insufficiencies[Title/Abstract])) OR (Renal Insufficiencies, Acute[Title/Abstract])) OR (Acute Kidney Insufficiency[Title/Abstract])) OR (Acute Renal Insufficiency[Title/Abstract])) OR (Kidney Insufficiency, Acute[Title/Abstract])) OR (Acute Kidney Insufficiencies[Title/Abstract])) OR (Kidney Insufficiencies, Acute[Title/Abstract])

#6 #4 OR #5

#7 "Cystatin C"[Mesh]

#8 (((((((((Cystatin 3[Title/Abstract]) OR (gamma-Trace[Title/Abstract])) OR (gamma Trace[Title/Abstract])) OR (Neuroendocrine Basic Polypeptide[Title/Abstract])) OR (Basic Polypeptide, Neuroendocrine[Title/Abstract])) OR (Post-gamma-Globulin[Title/Abstract])) OR (Post gamma Globulin[Title/Abstract])) OR (Serum cystatin C[Title/Abstract])) OR (Plasma cystatin C[Title/Abstract])) OR (Urinary cystatin C[Title/Abstract])

#9 #7 OR #8

#10 #3 OR #6 OR #9

Scopus

#1 ( TITLE-ABS-KEY ( Sepsis ) OR TITLE-ABS-KEY ( Bloodstream Infection ) OR TITLE-ABS-KEY ( Bloodstream Infections ) OR TITLE-ABS-KEY ( Infection , Bloodstream ) OR TITLE-ABS-KEY ( septicemia ) OR TITLE-ABS-KEY ( Septicemias ) OR TITLE-ABS-KEY ( Blood Poisoning ) OR TITLE-ABS-KEY ( Blood Poisonings ) OR TITLE-ABS-KEY ( Poisonings , Blood ) OR TITLE-ABS-KEY ( Poisoning , Blood ) OR TITLE-ABS-KEY ( Severe Sepsis ) OR TITLE-ABS-KEY ( Sepsis , Severe ) OR TITLE-ABS-KEY ( Pyemia ) OR TITLE-ABS-KEY ( Pyemias ) OR TITLE-ABS-KEY ( Pyaemia ) OR TITLE-ABS-KEY ( Pyohemia ) OR TITLE-ABS-KEY ( Pyohemias ) )

#2 ( TITLE-ABS-KEY ( Acute Kidney Injury ) OR TITLE-ABS-KEY ( Acute Kidney Injuries ) OR TITLE-ABS-KEY ( Kidney Injuries , Acute ) OR TITLE-ABS-KEY ( Kidney Injury , Acute ) OR TITLE-ABS-KEY ( Acute Renal Injury ) OR TITLE-ABS-KEY ( Acute Renal Injuries ) OR TITLE-ABS-KEY ( Renal Injuries , Acute ) OR TITLE-ABS-KEY ( Renal Injury , Acute ) OR TITLE-ABS-KEY ( Kidney Failure , Acute ) OR TITLE-ABS-KEY ( Acute Kidney Failures ) OR TITLE-ABS-KEY ( Kidney Failures , Acute ) OR TITLE-ABS-KEY ( Acute Kidney Failure ) OR TITLE-ABS-KEY ( Acute Renal Failure ) OR TITLE-ABS-KEY ( Acute Renal Failures ) OR TITLE-ABS-KEY ( Renal Failures , Acute ) OR TITLE-ABS-KEY ( Renal Failure , Acute ) OR TITLE-ABS-KEY ( Renal Insufficiency , Acute ) OR TITLE-ABS-KEY ( Acute Renal Insufficiencies ) OR TITLE-ABS-KEY ( Renal Insufficiencies , Acute ) OR TITLE-ABS-KEY ( Acute Kidney Insufficiency ) OR TITLE-ABS-KEY ( Acute Renal Insufficiency ) OR TITLE-ABS-KEY ( Kidney Insufficiency , Acute ) OR TITLE-ABS-KEY ( Acute Kidney Insufficiencies ) OR TITLE-ABS-KEY ( Kidney Insufficiencies , Acute ) )

#3 ( TITLE-ABS-KEY ( Cystatin C ) OR TITLE-ABS-KEY ( Cystatin 3 ) OR TITLE-ABS-KEY ( gamma-Trace ) OR TITLE-ABS-KEY ( gamma Trace ) OR TITLE-ABS-KEY ( Neuroendocrine Basic Polypeptide ) OR TITLE-ABS-KEY ( Basic Polypeptide , Neuroendocrine ) OR TITLE-ABS-KEY ( Post-gamma-Globulin ) OR TITLE-ABS-KEY ( Post gamma Globulin ) OR TITLE-ABS-KEY ( Serum cystatin C ) OR TITLE-ABS-KEY ( Plasma cystatin C ) OR TITLE-ABS-KEY ( Urinary cystatin C ) )

#4 #1 AND #2 AND #3

Web of science

#1 Sepsis (Topic) or Bloodstream Infection (Topic) or Bloodstream Infections (Topic) or Infection, Bloodstream (Topic) or Septicemia (Topic) or Septicemias (Topic) or Blood Poisoning (Topic) or Blood Poisonings (Topic) or Poisonings, Blood (Topic) or Poisoning, Blood (Topic) or Severe Sepsis (Topic) or Sepsis, Severe (Topic) or Pyemia (Topic) or Pyemias (Topic) or Pyaemia (Topic) or Pyohemia (Topic) or Pyohemias (Topic)

#2 Acute Kidney Injury (Topic) or Acute Kidney Injuries (Topic) or Kidney Injuries, Acute (Topic) or Kidney Injury, Acute (Topic) or Acute Renal Injury (Topic) or Acute Renal Injuries (Topic) or Renal Injuries, Acute (Topic) or Renal Injury, Acute (Topic) or Kidney Failure, Acute (Topic) or Acute Kidney Failures (Topic) or Kidney Failures, Acute (Topic) or Acute Kidney Failure (Topic) or Acute Renal Failure (Topic) or Acute Renal Failures (Topic) or Renal Failures, Acute (Topic) or Renal Failure, Acute (Topic) or Renal Insufficiency, Acute (Topic) or Acute Renal Insufficiencies (Topic) or Renal Insufficiencies, Acute (Topic) or Acute Kidney Insufficiency (Topic) or Acute Renal Insufficiency (Topic) or Kidney Insufficiency, Acute (Topic) or Acute Kidney Insufficiencies (Topic) or Kidney Insufficiencies, Acute (Topic)

#3 Cystatin C (Topic) or Cystatin 3 (Topic) or gamma-Trace (Topic) or gamma Trace (Topic) or Neuroendocrine Basic Polypeptide (Topic) or Basic Polypeptide, Neuroendocrine (Topic) or Post-gamma-Globulin (Topic) or Post gamma Globulin (Topic) or Serum cystatin C (Topic) or Plasma cystatin C (Topic) or Urinary cystatin C (Topic)

#1 AND #2 AND #3

Embase

#1 'sepsis'/exp OR 'sepsis'

#2 'bloodstream infection':ab,ti OR 'bloodstream infections':ab,ti OR 'infection, bloodstream':ab,ti OR 'septicemia':ab,ti OR 'septicemias':ab,ti OR 'blood poisoning':ab,ti OR 'blood poisonings':ab,ti OR 'poisonings, blood':ab,ti OR 'poisoning, blood':ab,ti OR 'severe sepsis':ab,ti OR 'sepsis, severe':ab,ti OR 'pyemia':ab,ti OR 'pyemias':ab,ti OR 'pyaemia':ab,ti OR 'pyaemias':ab,ti OR 'pyohemia':ab,ti OR 'pyohemias':ab,ti

#3 #1 OR #2

#4 'acute kidney failure'/exp OR 'acute kidney failure'

#5 'acute kidney injuries':ab,ti OR 'kidney injuries, acute':ab,ti OR 'kidney injury, acute':ab,ti OR 'acute renal injury':ab,ti OR 'acute renal injuries':ab,ti OR 'renal injuries, acute':ab,ti OR 'renal injury, acute':ab,ti OR 'kidney failure, acute':ab,ti OR 'acute kidney failures':ab,ti OR 'kidney failures, acute':ab,ti OR 'acute kidney failure':ab,ti OR 'acute renal failure':ab,ti OR 'acute renal failures':ab,ti OR 'renal failures, acute':ab,ti OR 'renal failure, acute':ab,ti OR 'renal insufficiency, acute':ab,ti OR 'acute renal insufficiencies':ab,ti OR 'renal insufficiencies, acute':ab,ti OR 'acute kidney insufficiency':ab,ti OR 'acute renal insufficiency':ab,ti OR 'kidney insufficiency, acute':ab,ti OR 'acute kidney insufficiencies':ab,ti OR 'kidney insufficiencies, acute':ab,ti

#6 #4 OR #5

#7 'cystatin c'/exp OR 'cystatin c'

#8 'cystatin 3':ab,ti OR 'gamma trace':ab,ti OR 'neuroendocrine basic polypeptide':ab,ti OR 'post gamma globulin':ab,ti OR 'cystatin c':ab,ti OR 'serum cystatin c':ab,ti OR 'plasma cystatin c':ab,ti OR 'urinary cystatin c':ab,ti

#9 #7 OR #8

#10 #3 AND #6 AND #9

sinomed

#1 “脓毒症”[不加权:扩展]

#2 “脓毒血症”[常用字段:智能] OR “脓血症”[常用字段:智能] OR “败血病”[常用字段:智能] OR "血液中毒"[常用字段:智能]

#3 (#2) OR (#1)

#4 “急性肾损伤”[不加权:扩展]

#5 “急性肾功能衰竭”[常用字段:智能] OR "急性肾脏功能衰竭"[常用字段:智能] OR "急性肾脏功能不全"[常用字段:智能] OR "肾功能不全,"[常用字段:智能] AND "急性"[常用字段:智能] OR "急性肾功能不全"[常用字段:智能】OR "肾功能衰竭,"[常用字段:智能] AND "急性"[常用字段:智能]

#6 (#5) OR (#4)

#7“胱抑素C"[常用字段:智能] OR“血清胱抑素C”[常用字段:智能] OR“血浆胱抑素C”[常用字段:智能】OR“尿液胱抑素C"[常用字段:智能]

#8 (#7) AND (#6) AND (#3)
