# Supplementary material for: Diagnostic value of cystatin C in acute kidney injury among patients with sepsis: a systematic review and meta-analysis
Source: Front Med (Lausanne). 2026 Jun 10;13:1769556. doi: 10.3389/fmed.2026.1769556 (PMC13290599; doi:10.3389/fmed.2026.1769556)

Supplement Legend

Supplementary Table 1 Characteristics of studies about other biomarkers included in the analysis.

Supplement figure 1 Risk of bias in included studies

Supplement figure 2 Fagan’s nomogram of the CysC for the prediction of SA-AKI.

Supplementary Table 1 Characteristics of studies about other biomarkers included in the analysis.

| Author | Year | Sepsis & AKI definition | No.  SA-AKI | No.  Non-AKI | Sample | Biomarker | AUC | Sen | Spe | Youden index | Cut-off value | |
| --- | --- | --- | --- | --- | --- | --- | --- | --- | --- | --- | --- | --- |
| Gou | 2024 | Sepsis3.0, KDIGO | 114 | 48 | Plasma | SOD  KIM‑1 | 0.7530  0.8220 | 0.5090  0.8070 | 0.9170  0.7290 | 0.4260  0.5360 | | 31.949 ng/mL  1.524 ng/L |
| Li | 2024 | Sepsis3.0, KDIGO | 82 | 216 | Blood | NGAL  BUN | 0.8670  0.7880 | 0.6944  0.6898 | 0.9512  0.7683 | 0.6456  0.4581 | | 93.210ng/mL  6.830mmol/L |
| Jing | 2024 | Sepsis3.0, KDIGO | 71 | 122 | Serum | P16INK4a  IL-37  P16INK4a+IL-37+ sCysC | 0.7650  0.8710  0.9160 | 0.7740  0.8028  0.9396 | 0.6960  0.7606  0.8310 | 0.6790  0.8240  0.8820 | | 1.535  101.460mmol/L  - |
| Yang | 2023 | Emergency treatment guidelines for sepsis/septic shock in China (2018) ^[26]^, KDIGO | 67 | 53 | Serum | HBP  PCT  HBP+PCT+ sCysC | 0.8270  0.8800  0.9670 | 0.7912  0.7613  0.9102 | 0.7740  0.9058  0.9247 | 0.5652  0.6671  0.8349 | | 36.265  5.205  - |
| Pei | 2022 | Sepsis3.0, KDIGO | 60 | 102 | Serum | SCr  NGAL  KIM-1  FGF-23  SCr +  sCysC | 0.8190  0.6200  0.7650  0.5960  0.8470 | 0.6010  0.3000  0.6170  0.5830  0.9500 | 0.9010  0.9310  0.8320  0.5940  0.7000 | 0.5020  0.2310  0.4490  0.1770  0.6500 | | 100.0μmol/L  95.600 ng/mL  135.700 pg/mL  322.100 pg/mL  - |
| Li | 2022 | Sepsis3.0, KDIGO | 74 | 158 | Serum | SCr  HDL-C  SCr+HDL-C+ sCysC | 0.6900  0.8900  0.9300 | 0.7660  0.8970  0.9550 | 0.7860  0.8860  0.9660 | 0.5520  0.7830  0.9210 | | 389.530μmol/L  0.630mmol/L  0.480 |
| Yi | 2022 | Surviving sepsis campaign: international guidelines for management of severe sepsis and septic shock: 2012, KDIGO | 51 | 66 | Serum | Scr  UmAlb  Scr+UmAlb+ sCysC | 0.6330  0.7560  0.9130 | 0.5690  0.6470  0.8820 | 0.7120  0.7580  0.8330 | 0.1280  0.4050  0.7150 | | 96.910(umol/L)  32.610(mg/24h)  - |
| Wei | 2021 | Sepsis3.0, KDIGO | 299 | 219 | Serum | Scr  APACHE П  APACHE Ⅱ +sCysC | 0.7300  0.9010  0.9350 | 0.6320  0.8260  0.8860 | 0.6940  0.8260  0.8540 | 0.3260  0.6520  0.7400 | | 74.750(umol/L)  13.5分  - |
| Wu | 2021 | Sepsis3.0, KDIGO | 40 | 62 | Serum | RBP  APACHEⅡ | 0.7920  0.7930 | 0.7250  0.7000 | 0.7260  0.8390 | 0.4510  0.5390 | | 77.500(mg/L)  23.510 |
| Bian | 2021 | Sepsis3.0, KDIGO | 29 | 51 | Serum | Scr | 0.6520 | NP | NP | - | | NP |
| Wu | 2020 | Sepsis3.0, KDIGO | 112 | 93 | Serum | Scr  uNGAL  uNGAL+ sCys-C | 0.8200  0.8970  0.9840 | 0.7910  0.8730  0.9550 | 0.7530  0.8020  0.9630 | 0.5440  0.6750  0.9180 | | NP  231.100g/L  NP |
| Zhu | 2019 | Sepsis3.0, KDIGO | 27 | 49 | Blood | Scr | 0.5920 | NP | NP | - | | NP |
| Liu | 2019 | 2012 SSC Guidelines for Sepsis, KDIGO | 42 | 47 | Serum | NGAL  KIM-1  NGAL+KIM-1+ sCysC | 0.7760  0.7850  0.8910 | 0.7500  0.8100  0.9300 | 0.7900  0.7800  0.8700 | 0.5400  0.5900  0.8000 | | 16.320  1.870  - |
| Chi | 2018 | Surviving sepsis campaign: international guidelines for management of severe sepsis and septic shock: 2012; KDIGO | 72 | 66 | Serum | APACHEⅡ  sCysC+APACHEⅡ | 0.8070  08800 | 0.5970  0.7080 | 0.8940  0.9240 | 0.4510  0.6320 | | 25.000  - |
| Yang | 2017 | NP; KDIGO | 48 | 57 | Serum | Scr  IGFBP-7 | 0.6290  0.8810 | 0.5420  0.9380 | 0.7190  0.7720 | 0.2610  0.7100 | | NP  1.820ng/mL |
| Zhou | 2016 | 2012 SSCM Guidelines for Sepsis, KDIGO | 18 | 20 | Serum | uIL-18  sCys-C+uIL-18 | 0.7850  0.9400 | 0.8270  NP | 0.7500  NP | 0.5770  - | | 265.000ng/L |
| Dai | 2015 | the 2001 International Sepsis Definition Conference; KDIGO | 55 | 57 | Plasma/ urine | pNGAL  uNGAL  psTREM-1  usTREM-1 | 0.8230  0.8550  0.7940  0.7070 | NP  NP  NP  NP | NP  NP  NP  NP | -  -  -  - | | NP  NP  NP  NP |

AUC, area under the curve of receiver operator characteristics. Sen, sensitivity. Spe, specificity. RBP, Retinol Binding Protein.NP, not report. SOD, Superoxide Dismutase.KIM-1,Kidney Injury Molecule-1. BUN, Blood urea nitrogen. IL-37,interleukin-37. HBP ,Heparin-binding protein.PCT, procalcitonin.

HDL-C,High density lipoprotein cholesterol. APACHEⅡ,Acute physiology and chronic health evaluation Ⅱ.SCr,Serum creatinine. uIL-18, urine interleukin-18.IGFBP-7,Insulin-like growth factor binding protein-7.pNGAL, plasma Neutrophil gelatinase-associated lipocalin. uNGAL, urine Neutrophil gelatinase-associated lipocalin. psTREM-1, plasma soluble triggering receptor expressed on myeloid cells-1. usTREM-1, urine soluble triggering receptor expressed on myeloid cells-1.

Supplement figure 1 Risk of bias in included studies


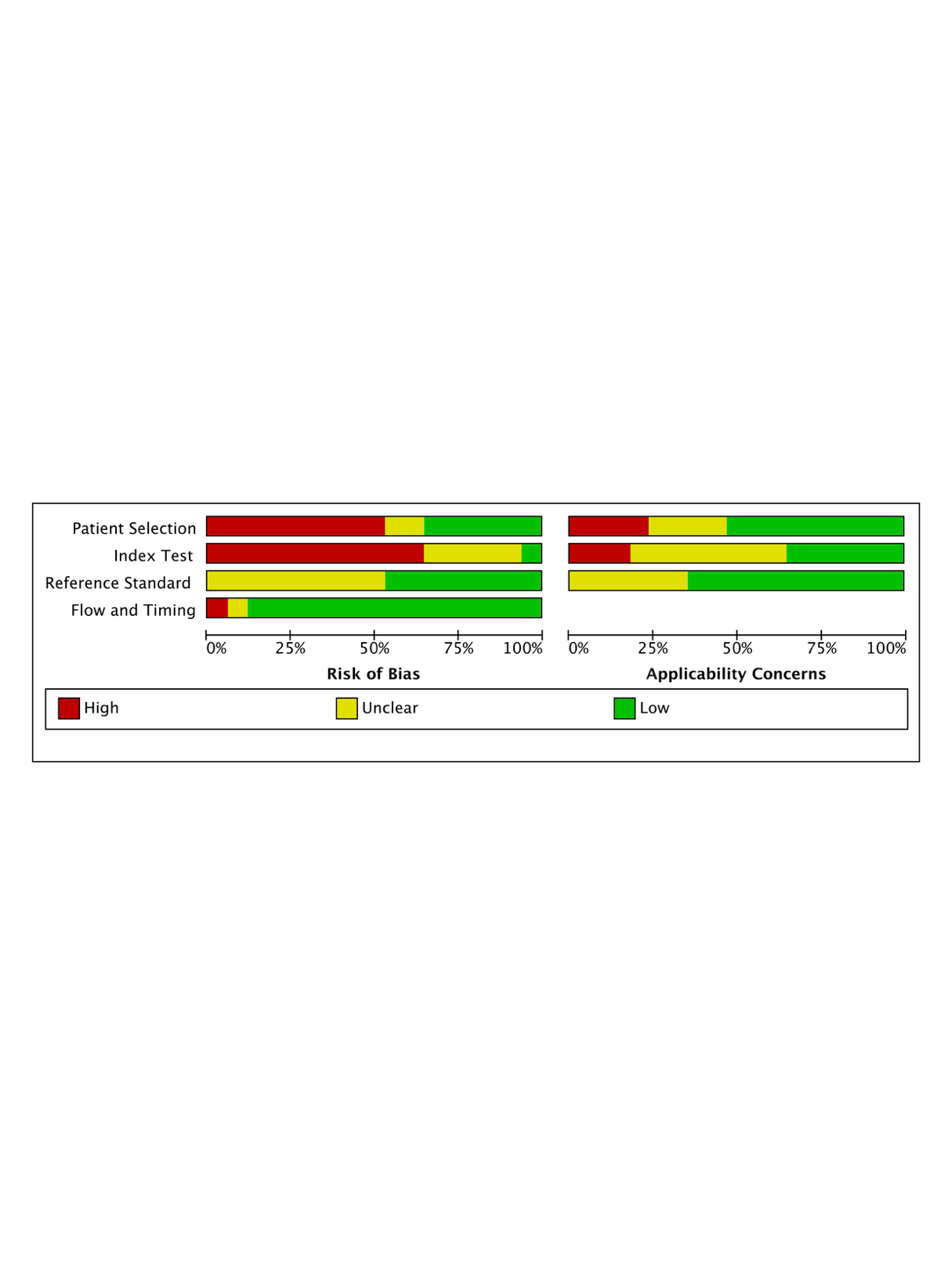


Supplement figure 2 Fagan’s nomogram of the CysC for the prediction of SA-AKI.


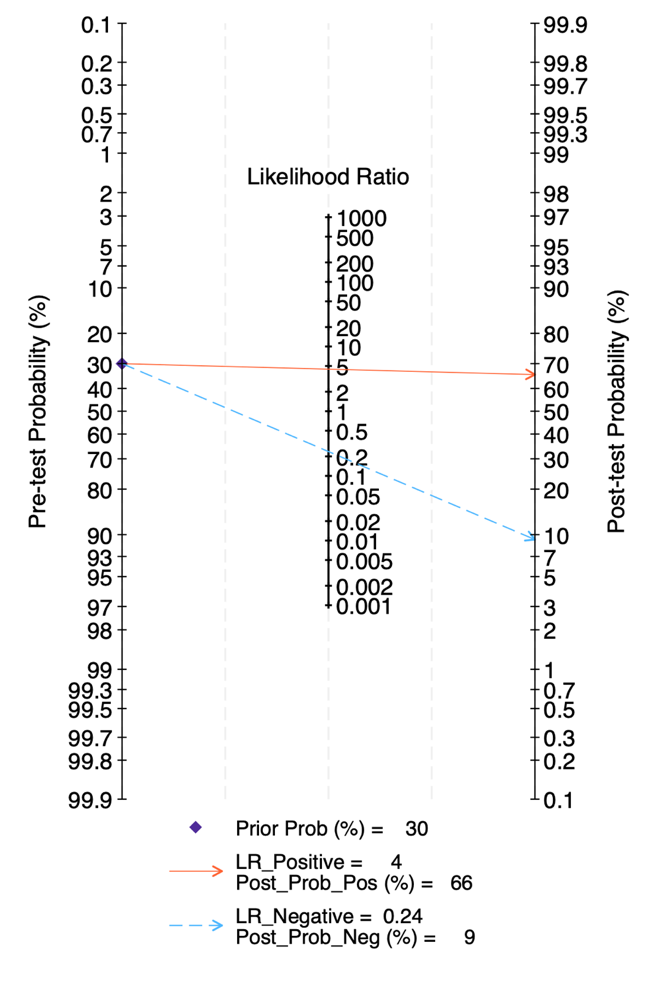

Supplement: Supplementary file 3 [file Table_1.DOCX]
