# Supplementary material for: Diagnostic value of cystatin C in acute kidney injury among patients with sepsis: a systematic review and meta-analysis
Source: Front Med (Lausanne). 2026 Jun 10;13:1769556. doi: 10.3389/fmed.2026.1769556 (PMC13290599; doi:10.3389/fmed.2026.1769556)
Supplement: Supplementary file 4 [file Table_2.DOCX]

Supplement Legend

Supplement figure 3 Diagnostic accuracy for Cys-C of research before year 2019

Supplement figure 4 Diagnostic accuracy for Cys-C of research after year 2019

Supplement figure 5 Diagnostic accuracy for Cys-C of research sample size greater than 100

Supplement figure 3 Diagnostic accuracy for Cys-C of research before year 2019

**
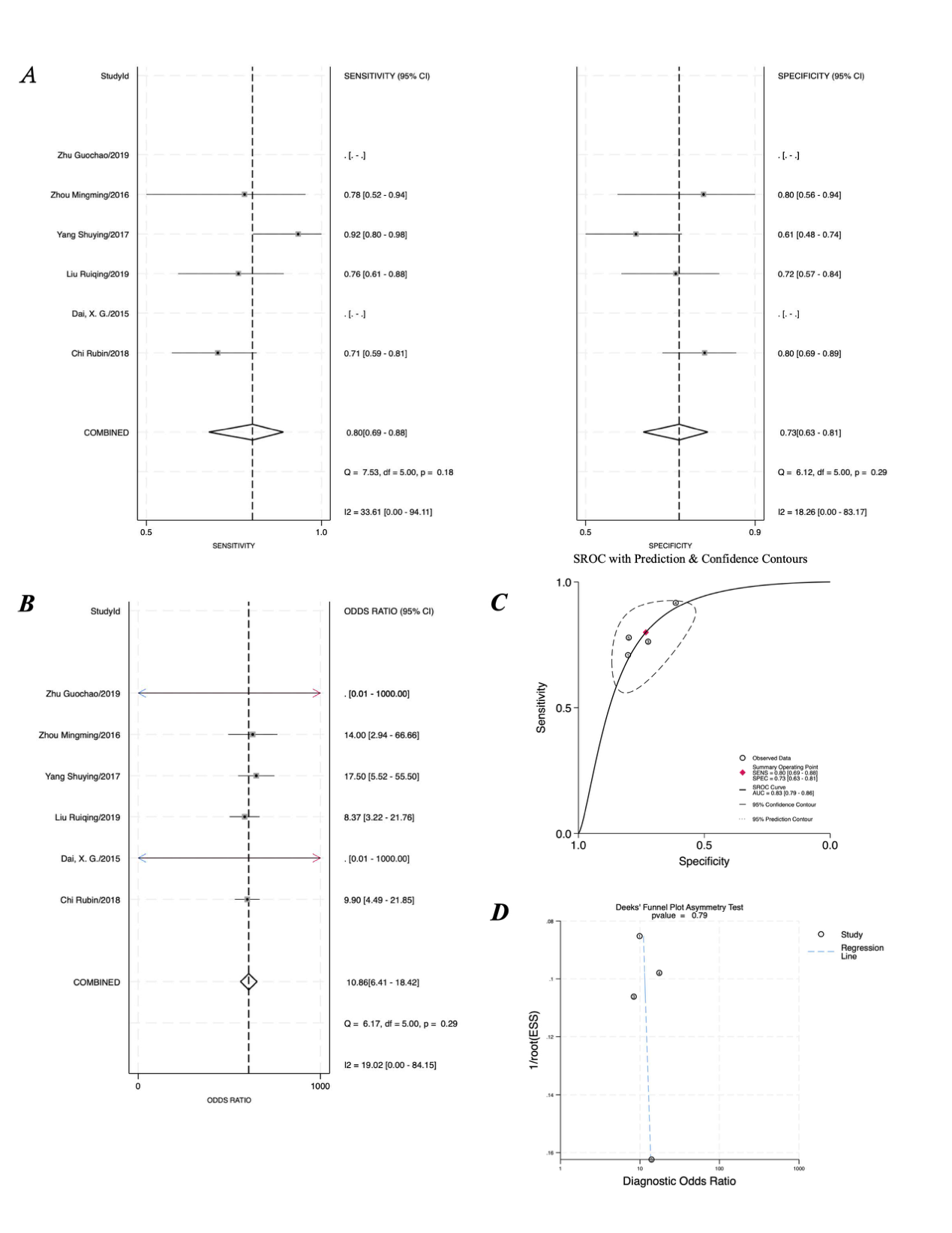
**

(A) Diagnostic sensitivity and specificity (B) Diagnostic accuracy (C) Receiver operating characteristic curve (ROC) (D) Publication bias.

Supplement figure 4 Diagnostic accuracy for Cys-C of research after year 2019

**
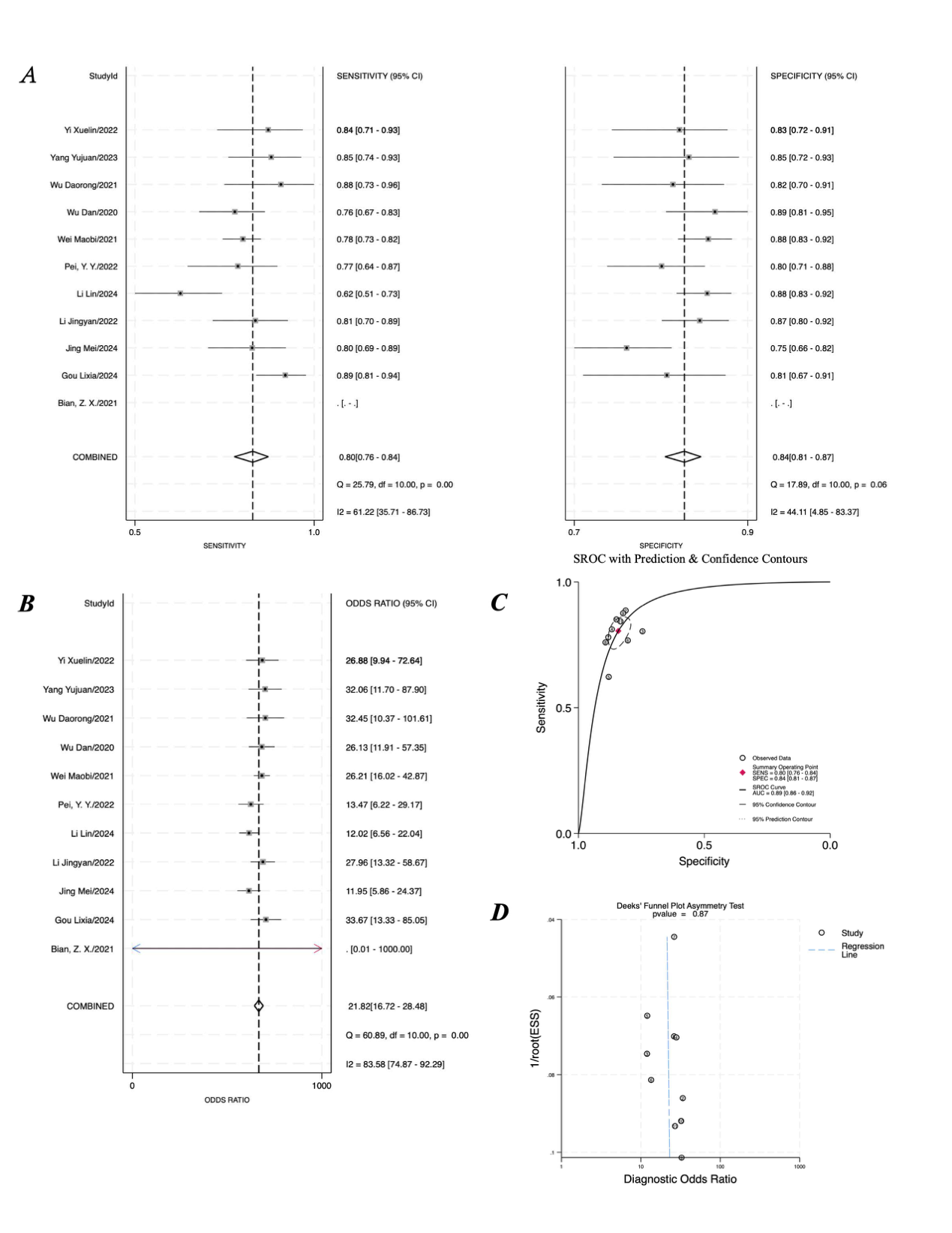
**

(A) Diagnostic sensitivity and specificity (B) Diagnostic accuracy (C) Receiver operating characteristic curve (ROC) (D) Publication bias.

Supplement figure 5 Diagnostic accuracy for Cys-C of research sample size greater than 100


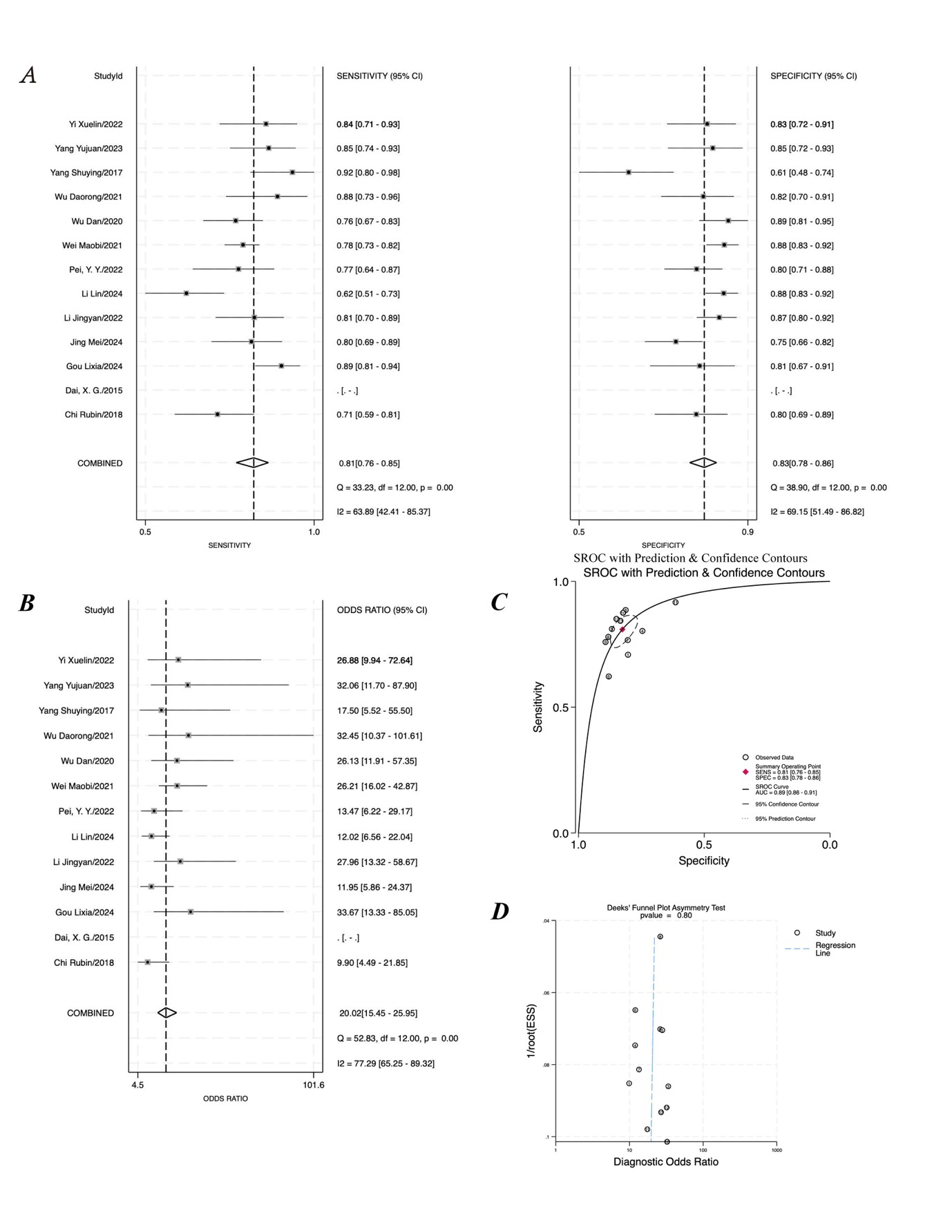
(A) Diagnostic sensitivity and specificity (B) Diagnostic accuracy (C) Receiver operating characteristic curve (ROC) (D) Publication bias.
