# Supplementary material for: Diagnostic value of cystatin C in acute kidney injury among patients with sepsis: a systematic review and meta-analysis
Source: Front Med (Lausanne). 2026 Jun 10;13:1769556. doi: 10.3389/fmed.2026.1769556 (PMC13290599; doi:10.3389/fmed.2026.1769556)
Supplement: Supplementary file 5 [file Table_3.DOCX]

Supplement Legend

Supplement figure 6 Diagnostic accuracy for Cys-C of research sample size less than 100

Supplement figure 7 Diagnostic accuracy for Cys-C with a cut-off value >1.5 mg/L.

Supplement figure 8 Diagnostic accuracy for Cys-C with a cut-off value < 1.5 mg/L

Supplement figure 6 Diagnostic accuracy for Cys-C of research sample size less than 100

**
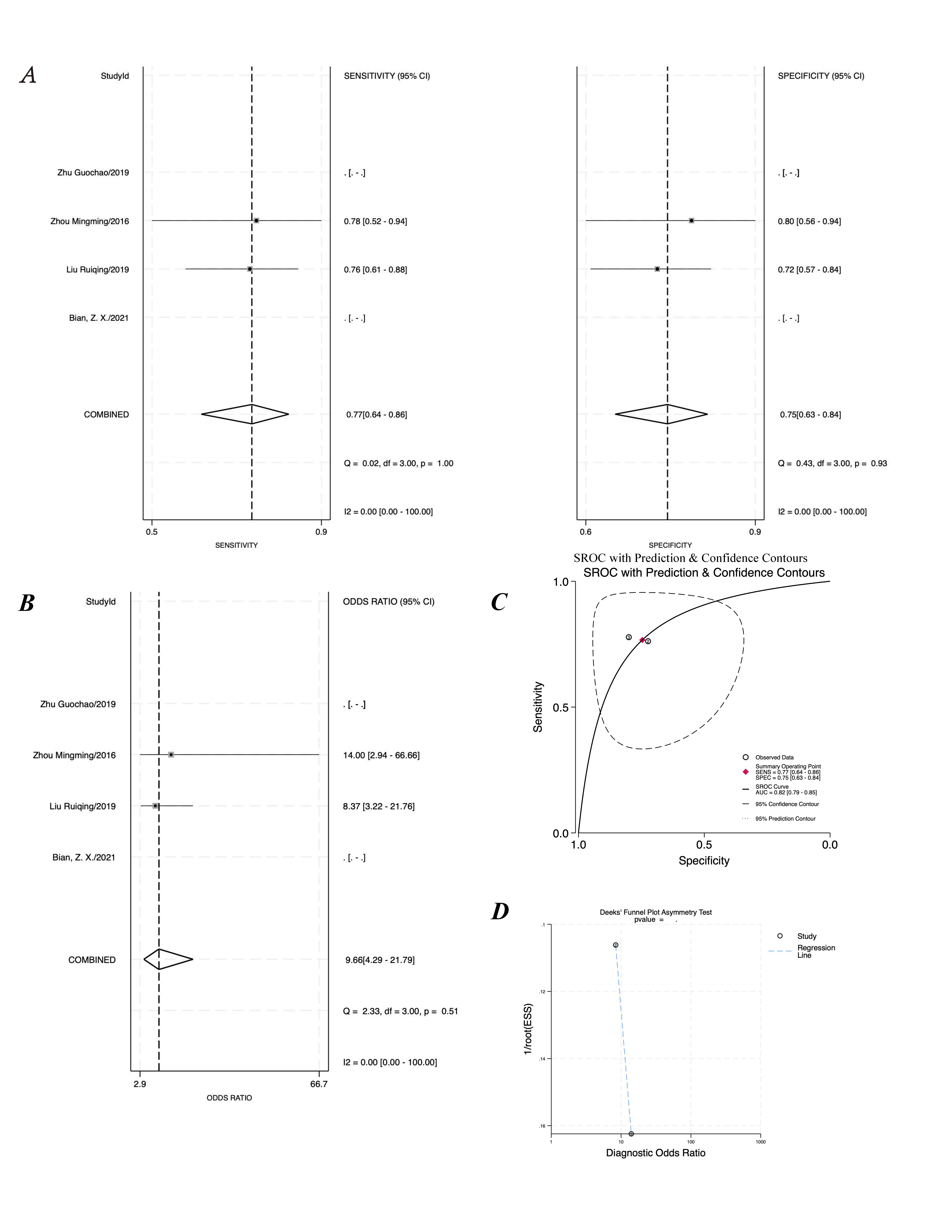
**

(A) Diagnostic sensitivity and specificity (B) Diagnostic accuracy (C) Receiver operating characteristic curve (ROC) (D) Publication bias.

Supplement figure 7 Diagnostic accuracy for Cys-C with a cut-off value >1.5 mg/L.


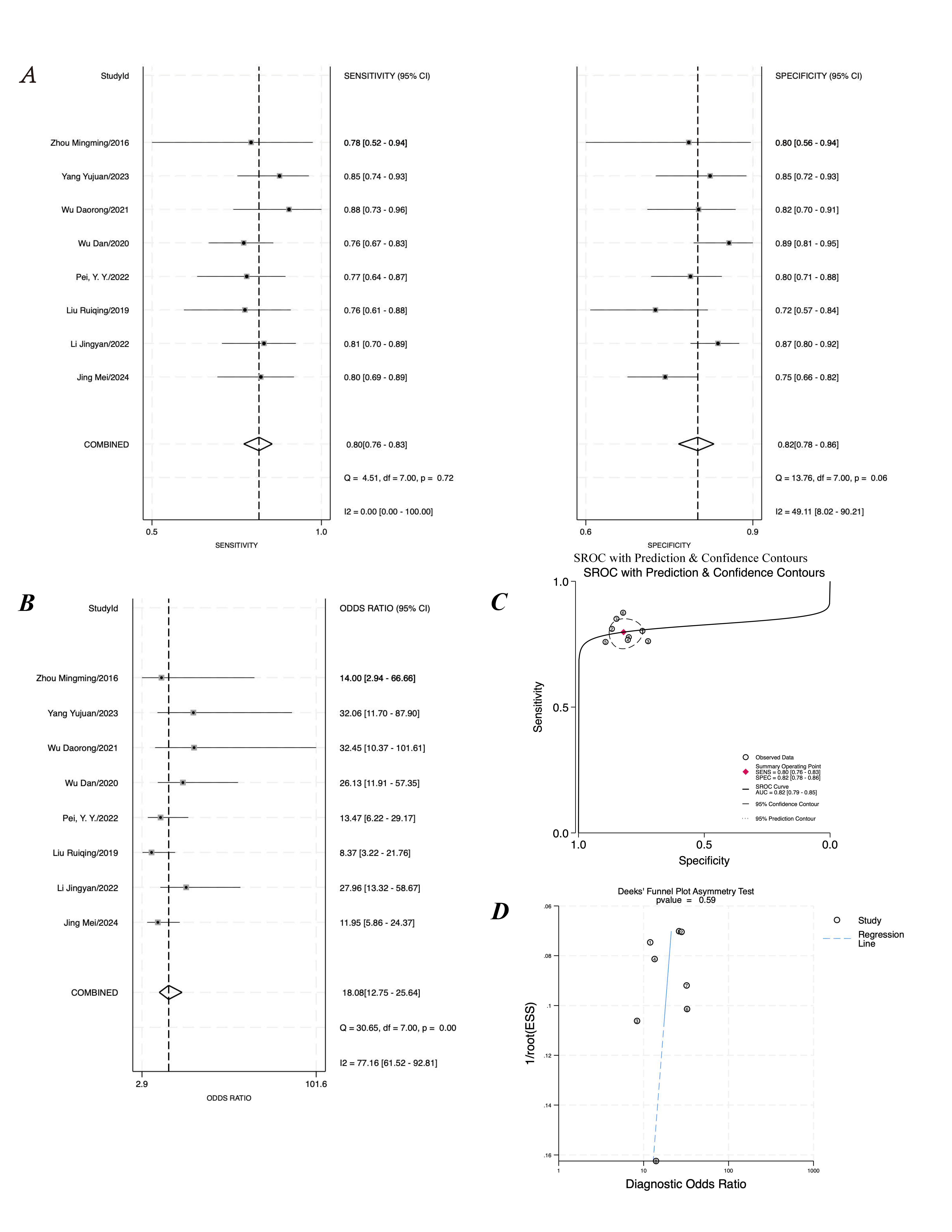


(A) Diagnostic sensitivity and specificity (B) Diagnostic accuracy (C) Receiver operating characteristic curve (ROC) (D) Publication bias.

Supplement figure 8 Diagnostic accuracy for Cys-C with a cut-off value < 1.5 mg/L


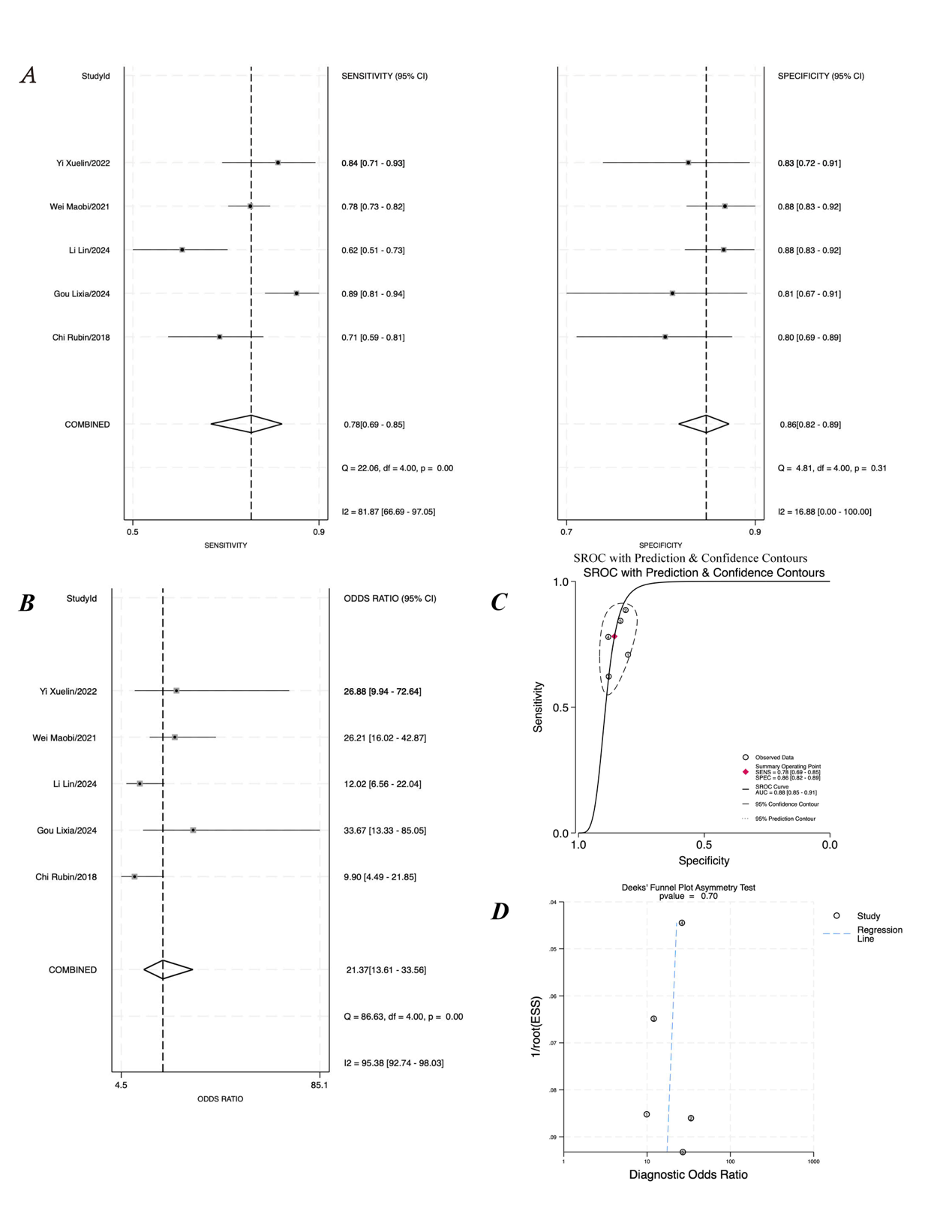


(A) Diagnostic sensitivity and specificity (B) Diagnostic accuracy (C) Receiver operating characteristic curve (ROC) (D) Publication bias.z
